# Osteogenic potential of human adipose derived stem cells (hASCs) seeded on titanium trabecular spinal cages

**DOI:** 10.1038/s41598-020-75385-y

**Published:** 2020-10-26

**Authors:** Laura Caliogna, Valentina Bina, Laura Botta, Francesco Maria Benazzo, Marta Medetti, Gianluca Maestretti, Mario Mosconi, Fabio Cofano, Fulvio Tartara, Giulia Gastaldi

**Affiliations:** 1 Orthopedics and Traumatology Clinic, IRCCS Policlinico San Matteo Foundation, 27100 Pavia, Italy; 2 Department of Biology and Biotechnology “Lazzaro Spallanzani”, University of Pavia, 27100 Pavia, Italy; 3 Department of Clinical Surgical, Diagnostic and Pediatric Sciences, University of Pavia, 27100 Pavia, Italy; 4 Centre for Health Technologies, University of Pavia, 27100 Pavia, Italy; 5 Department of Molecular Medicine, University of Pavia, 27100 Pavia, Italy; 6 Istituto Clinico Città Studi, Milan, Italy; 7 Department of Orthopaedic Surgery, Cantonal Hospital, 1708 Fribourg, Switzerland; 8 Unit of Neurosurgery, Department of Neuroscience, “Rita Levi Montalcini”, 10126 Turin, Italy; 9 Spine Surgery Unit, Humanitas Gradenigo, Turin, Italy

**Keywords:** Biotechnology, Cell biology, Materials science

## Abstract

Spine degenerative conditions are becoming increasingly prevalent, affecting about 5.7% of the population in Europe, resulting in a significant reduction of life’s quality. Up to now, many materials have been used in manufacturing cage implants, used as graft substitutes, to achieve immediate and long-term spinal fixation. Particularly, titanium and its alloys are emerging as valuable candidates to develop new types of cages. The aim of this in vitro study was to evaluate the adhesion, proliferation and osteogenic differentiation of adipose derived mesenchymal stem cells (ASCs) seeded on trabecular titanium cages. ASCs adhered, proliferated and produced an abundant extracellular matrix during the 3 weeks of culture. In the presence of osteogenic medium, ASCs differentiated into osteoblast-like cells: the expression of typical bone genes, as well as the alkaline phosphatase activity, was statistically higher than in controls. Furthermore, the dispersive spectrometry microanalysis showed a marked increase of calcium level in cells grown in osteogenic medium. Plus, our preliminary data about osteoinduction suggest that this titanium implant has the potential to induce the ASCs to produce a secretome able to trigger a shift in the ASCs phenotype, possibly towards the osteogenic differentiation, as illustrated by the qRT-PCR and ALP biochemical assay results. The trabecular porous organization of these cages is rather similar to the cancellous bone structure, thus allowing the bone matrix to colonize it efficiently; for these reasons we can conclude that the architecture of this cage may play a role in modulating the osteoinductive capabilities of the implant, thus encouraging its engagement in in vivo studies for the treatment of spinal deformities and diseases.

## Introduction

Degenerative conditions of the cervical and lumbar spine are becoming increasingly prevalent, resulting in significant reduction of quality of life caused by pain^1^.

Spinal fusion procedures were initially employed to treat these pathologies by the use of structural autografts and allografts, but they were associated with intra- and post-operative complications and vertebral non-union. Spinal instrumentation (anterior and posterior) was later introduced to enhance spinal stability, while restoring spine balance^2^.

In the 1950s the fusion was obtained by the use of a bone graft. It was collected from the iliac crest or via allograft bone bank, then it was pre-cutted and inserted through distraction and force^3^.

However, this technique was associated with a high level of morbidity as pain, wound drainage, infection, haematomas, nerve injury and iliac crest fractures or deformity^4^.

In order to limit the morbidity of these procedures, new types of implants have been developed. Cages were introduced and used as graft substitutes. For the first time Bagby attempted to use these structures in horses, proposing the cage fusion technology: a cylindrical fenestrated and hollow device made of stainless steel to permit bone ingrowth^5^.

The construct became biologically integrated into the native spine, and living bone replaced the implant over time. This device was soon adapted to humans for lumbar and cervical spine and further new implant design and material were developed to improve clinical outcomes^6^.

An ideal cage design has to ensure the disc height and the correct alignment, reducing complication rates, but it also has to achieve an immediate postoperative stability to guarantee the osteointegration of the implant and its long-term fixation. Basic cage type is a small implant with lateral, upper and/or lower windows and a central cavity filled with bone or osteo-inductive materials^7^. In fact, they are also employed as carriers for osteo-inductive or osteo-conductive agents to ensure long-term stability through biologic integration^8^.

Main goal of the these treatments is to achieve the spine fusion, stabilizing the motion segment, while restoring the correct alignment^9,10^.

The need to generate stable spine alignment, correcting deformities, has also brought attention to the use of materials able to guarantee primary stability and biological integration capacity. Many materials had been used in the manufacture of these implants and the evolution of cage materials has accompanied changes in design. Particularly, cages made of highly porous materials have been used in the last years in order to treat lumbar degenerative disease with sagittal imbalance^11^.

Due to its properties, titanium and its alloys were used to develop new types of cages to reduce complication rates by promoting early osteointegration through modification of cage surfaces^12^.

The cage utilized in this study had upper and lower surfaces made out of trabecular titanium (TT) in order to assure an optimal ground for bone ingrowth. The scaffold had also a later oval window to allow the insertion of a bone graft, enhancing the osteo-integration of the implant.

Some custom-made trabecular titanium cages have been already used for patients affected by spinal deformity and sagittal imbalance by the acquisition of a 2D high-resolution CT scan, improving the correction of deformities and restoring the patient specific sagittal alignment^13^.

The aim of this study was to evaluate in vitro the adhesion and proliferation of cells on trabecular titanium cages. Moreover, we analyzed the osteoblastic differentiation of adipose derived mesenchymal stem cells isolated from human subcutaneous tissue (hASCs), grown on these scaffolds and in monolayer, while cultured with growth, conditioned and osteogenic media (GM, CM, OM). We evaluated the differentiation of hASCs by analyzing some known indicators of the osteoblast phenotype: alkaline phosphatase activity (ALP), reverse transcriptase RT-PCR of alkaline phosphatase (*alp*), *runx-2* and *ibsp*. Furthermore, we performed scanning electron microscopy (SEM) to evaluate the presence of extracellular matrix (ECM) and X-ray microanalysis for detection of calcified extracellular matrix.

## Materials and methods

### Scaffolds

The cages used in this in vitro study were given by MT Ortho company (MT Ortho s.r.l., Aci Sant'Antonio, CT, Italy). The cage was made out of porous titanium (Ti6Al4V) modeled by CAD/CAM technology by means of electron beam melting (EBM) technology. The average diameter of pores was 0.810 ± 0.067 mm.

### Human adipose derived stem cells (hASCs)

Subcutaneous adipose tissue was obtained from healthy donors during hip replacement surgery. The age range was 60–70 years and the body mass index was 22.5–26.5. The study was conducted in accordance with the 1975 Declaration of Helsinki; informed consent was obtained from all patients before surgery and the protocol was approved by Ethics Committee of San Matteo Foundation, Research and Care Institute, Pavia, Italy (P-20190023312, 9th April 2019). The specimens preserved in sterile conditions were transported to the laboratory for processing. In brief, the tissue finely minced was incubated in digestion buffer (0.01% collagenase type II in DMEM F12-HAM medium) for 1 h at 37 °C in a shaking water bath^14^. At the end of the incubation time, the collagenase was neutralized and the suspension filtered (membrane pore size: 100 µm) to remove the debris and centrifuged at 1200 rpm for 10 min at 4 °C. The pellet was washed twice, treated with lysis solution and finally suspended in growth medium (GM, DMEM F12-HAM supplemented with 10% FBS, 100U/ml penicillin, 100 μg/ml streptomycin and 0.25 µg/ml amphotericin). The hASCs were cultured in GM up to 95% confluence in a humidified atmosphere of 95% air with 5% CO2 at 37 °C. The adherent cells were trypsinized with Trypsin EDTA, and 5000 hASCs/cm^2^ tissue culture plate were seeded in a new flask. These passages were repeated three times. At the third passage, the hASCs were positive for the mesenchymal stem cell markers CD73, CD90, and CD105 and negative for the hematopoietic cell markers CD34 and CD45 according to the analysis performed by flow cytometer (Navios Beckman Coulter). Data were acquired, displayed and elaborated by Kaluza 1.2 software package (Beckman Coulter). The positive cells were counted and compared with the signal of corresponding immunoglobulin isotypes^15^.

### Cell seeding and culture

At the third passage cells were trypsinized and 200,000 cells were seeded onto each cage placed inside a 12 well-plate. The cellular suspension was allowed to be adsorbed by the porous substrates in a humidified atmosphere of 95% air with 5% CO_2_ at 37 °C. After 2 h, GM was added to cover the cell/cage construct.

### Adhesion and proliferation

To evaluate the mitochondrial activity of the seeded cells on scaffolds during the culture period, a test with 3-(4, 5-dimethylthiazole-2-yl)-2,5-diphenyl tetrazolium bromide (MTT) was performed on days 1, 7, 14, and 21. To exclude the signal arisen from cells adhered on the plastic plates, the scaffolds were moved in new wells before the MTT assay was carried out. The scaffolds were soaked in a 0.5 mg/mL solution of MTT in PBS, and the cell/cage constructs were incubated for 4 h in a humidified atmosphere of 95% air with 5% CO_2_ at 37 °C. Viable cells are able to reduce MTT into formazan crystals. After removing the MTT solution, to solubilize the formazan products, 1 mL of acidic isopropanol was added to each cell/cage construct. Absorbance values were read at 570 nm. An appropriate standard curve was made for each experiment.

### Osteogenic differentiation

After the seeding of hASCs all the hASC/cages were cultured with GM for 7 days before the induction of osteogenic differentiation. For induction of osteogenic differentiation, half of the constructs were switched into osteogenic medium (OM; DMEM F12-HAM containing 15% FBS, 10 mM betaglycerophosphate, 100 nM dexamethasone, 0.05 mM ascorbic acid, antibiotics, and amphotericin). The other constructs (controls) were maintained in GM. To investigate the osteoinductive capabilities of these cages, hASCs were grown in monolayers in polystyrene wells and cultured with growth, conditioned and osteogenic media. CM was obtained by mixing up 50% of fresh GM with GM enriched with the secretome derived from hASCs cultured on titanium scaffolds without osteogenic factors. Both qRT-PCR and ALP assay were carried out at 7, 14 and 21 days of in vitro culture, as explained below.

### RNA isolation and reverse transcriptase quantitative real-time PCR (qRT-PCR)

To evaluate gene expression after 14 days from the induction of osteogenic differentiation, RNA was extracted from the constructs with QIAzol Lysis Reagent. The total RNA extracted was reverse-transcribed into cDNA using random hexamers and M-MLV Reverse Transcriptase, according to Laforenza et al. 2010^16^. Quantitative real-time RT-PCR was performed in triplicate using 1 µL cDNA obtained as above, using specific primers from Qiagen: ALP (QT00012957), RUNX-2 (QT00020517) and ISBP (QT00093709). Quantifast-SYBR Green PCR Kit (Qiagen) was used according to the manufacturer’s instruction and qPCR was performed using Rotor Gene 6000 (Corbett). Cycling conditions: initial denaturation at 95 °C for 5 min; 40 cycles of denaturation at 95 °C for 30 s; annealing at 60 °C for 30 s, and elongation at 72 °C for 40 s. Melting curves were generated to identify the melting temperatures of specific products after the PCR run. The qPCR reactions were normalized against the expression of the housekeeping gene GUSB (beta-glucuronidase, QT00046046, Qiagen). Results of gene expression of differentiated cells were expressed as fold change versus expression of hASC on scaffold after 7 days culture in growth medium.

### Alkaline phosphatase (ALP) activity

To confirm osteogenic differentiation, ALP activity was evaluated at days 7, 14 and 21 after the induction of osteogenic differentiation. To exclude the signal arisen from cells adhered on the plastic plates, the scaffolds were moved in new wells before the ALP assay was carried out. The culture medium was removed and the scaffolds were rinsed in PBS and incubated with 5 mM p-nitrophenyl-phosphate in 50 mM glycine, 1 mM MgSO4, 1 mM ZnSO4, pH 10.5 at 37 °C for 10 min. The absorbance of p-nitrophenol formed was read at 410 nm. The ALP activity of hASC/cages was expressed as the formation of 1 nmol p-nitrophenol/10 ^5^ cells x min; instead the ALP activity of hASCs grown in monolayer was expressed as 1 nmol p-nitrophenol/ mg prot × 15 min.

### SEM analysis

For electron microscopy evaluation, cells/cage constructs after 21 days from differentiation were washed with PBS then fixed with glutaraldeyde 2.5% in 0.4 M of Sodium Cacodylate Buffer for 2 h and 30 min. Then the constructs were washed with Sodium Cacodylate buffer for 30 min and dehydrated with graded ethanol series, starting with 50, 70, 90 and 100%.

Microstructural characterization was performed with a high-resolution scanning electron microscope (SEM: EVO 40 SMART scanning electron microscope, Zeiss and TESCAN Mira 3 XMU) operated at 20 kV. The composition microanalysis was determined by energy dispersive spectrometry (EDS, EDAX). The samples were previously coated with carbon using a Cressington carbon coater 208c.

SEM–EDS analyses were performed at Arvedi Laboratory, CISRiC (Centro Interdipartimentale di Studi e Ricerche per la Conservazione del Patrimonio Culturale), University of Pavia.

### Statistics

Data are presented as mean ± SEM of four different preparations. Statistical analyses were performed using the one-way ANOVA method followed by Newman-Keuls’ Q test (Graph-Pad Prism 4.0).

## Results

### Adhesion and proliferation of hASCs

**Figure 1:**
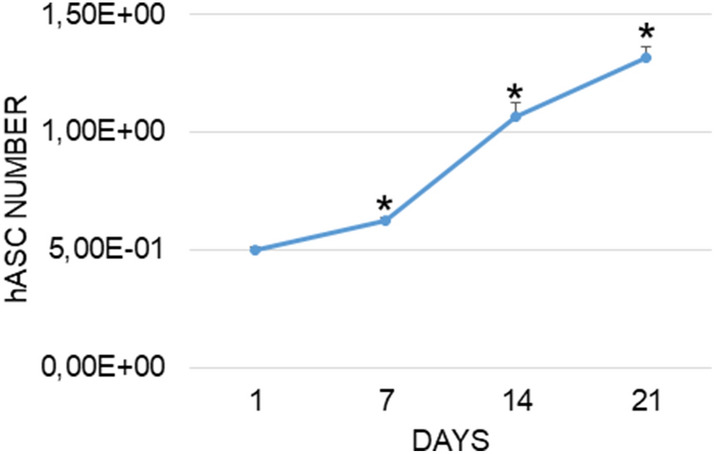
hASC proliferation after seeding on cage. Each point is the mean ± SEM of 4 different experiments. When not present the standard error was within the symbol. *, p ≤ 0.05 versus 1 day (one-way ANOVA method followed by Newman-Keuls’Q test).

MTT test showed that hASCs seeded on the cages adhered and proliferated during the 3 weeks of culture. After 7 days of culture, the hASCs number was already statistically higher than that of day 1 (Fig. 1).

### SEM analysis

**Figure 2:**
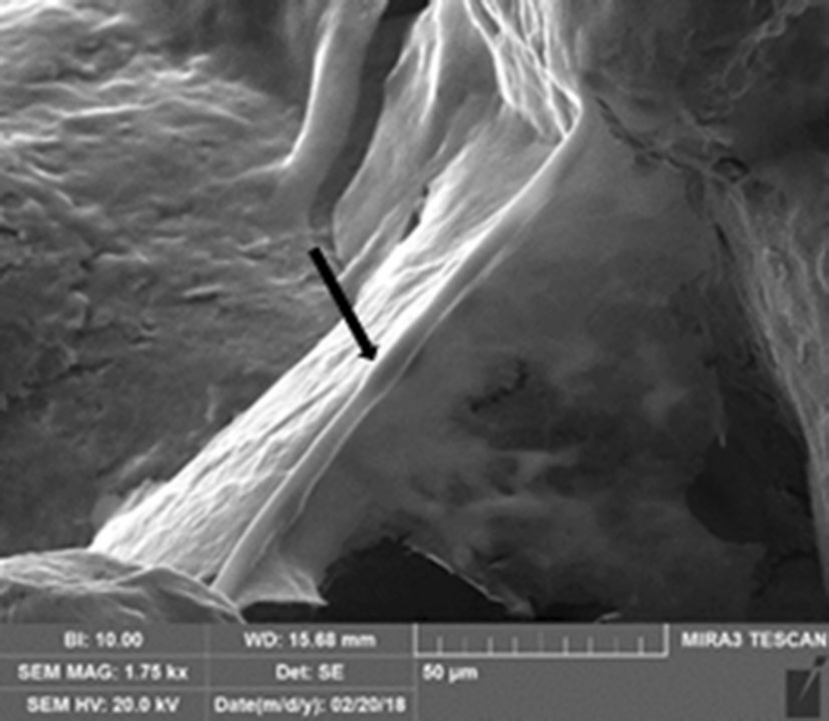
SEM analysis of a cage cultured in osteogenic medium. The arrow highlights the presence of a little capillary. Magnification: 1, 75 kx.

Empty cage showed a rough surface with a regular porous structure (Supplementary Figure S1A). The cells/cage constructs showed the presence of many cells and an abundant extracellular matrix, and both in growth medium (Supplementary Figure S1B) and osteogenic medium (Supplementary Figure S1C), the matrix fills the pores. The internal side of the cage showed a structure identical with respect to the outer side (Supplementary Figure S2A). The cells have migrated inward and secreted extracellular matrix more evidently in cages cultured in osteogenic medium (Supplementary Figure S2, B and C). At higher magnification, on the cage was evident also a tubular structure that could be a capillary (Fig. 2, arrow).

**Figure 3:**
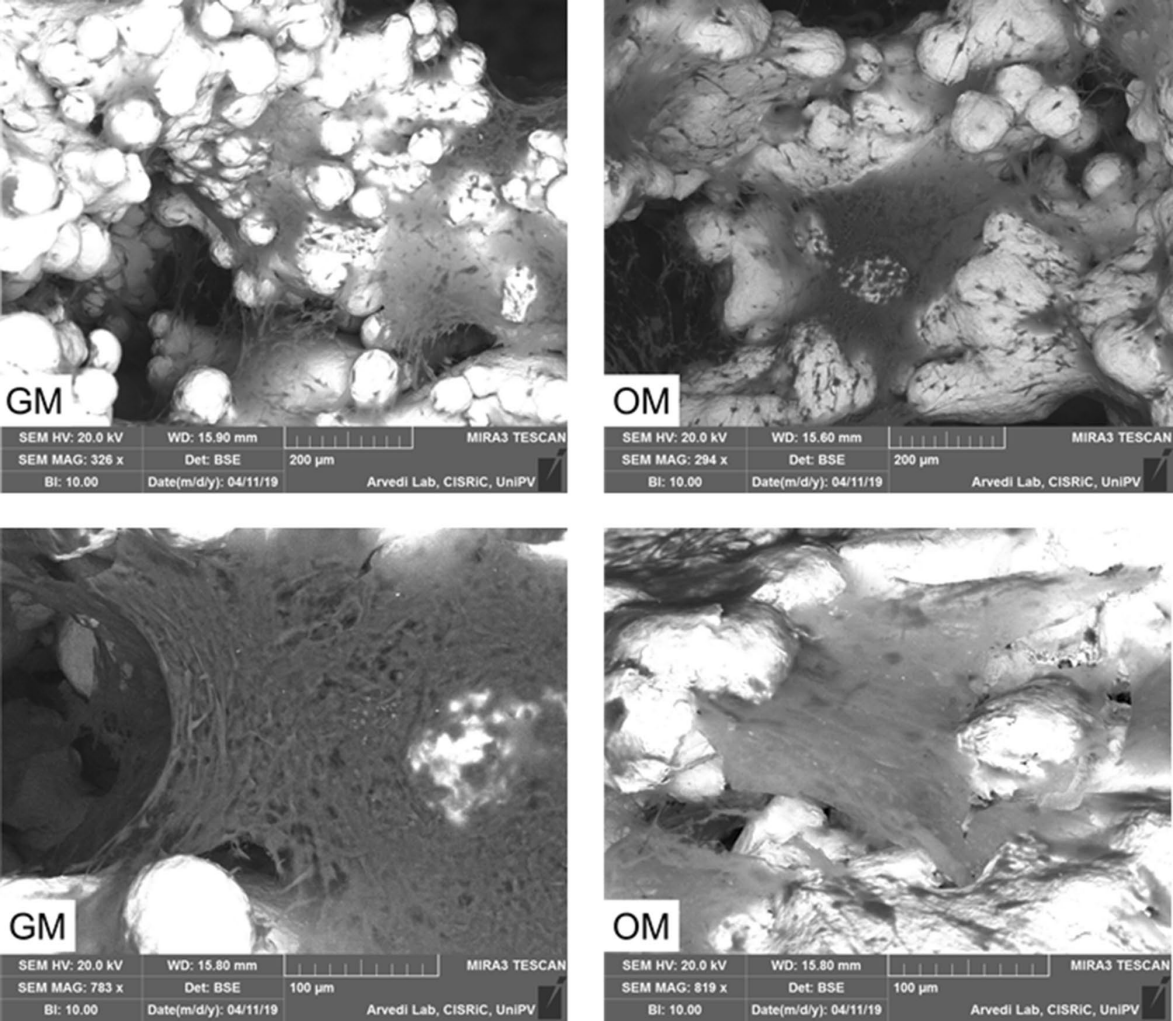
SEM analysis of hASC/cage constructs cultured with growth (GM) and osteogenic (OM) medium. The BSE detection showed the organic parts (cells and their matrix) that appeared dark, while the metallic scaffold appeared clear. The matrix of constructs in OM appeared thicker.

The BSE (back scattered electrons) detector allowed to better visualize cells and their own matrix that appeared dark, while the metallic scaffold appeared clear (Fig. 3). Again, what emerged is that there was more matrix on constructs cultured in osteogenic medium than in controls.

### Osteogenic differentiation

#### mRNA expression

**Figure 4:**
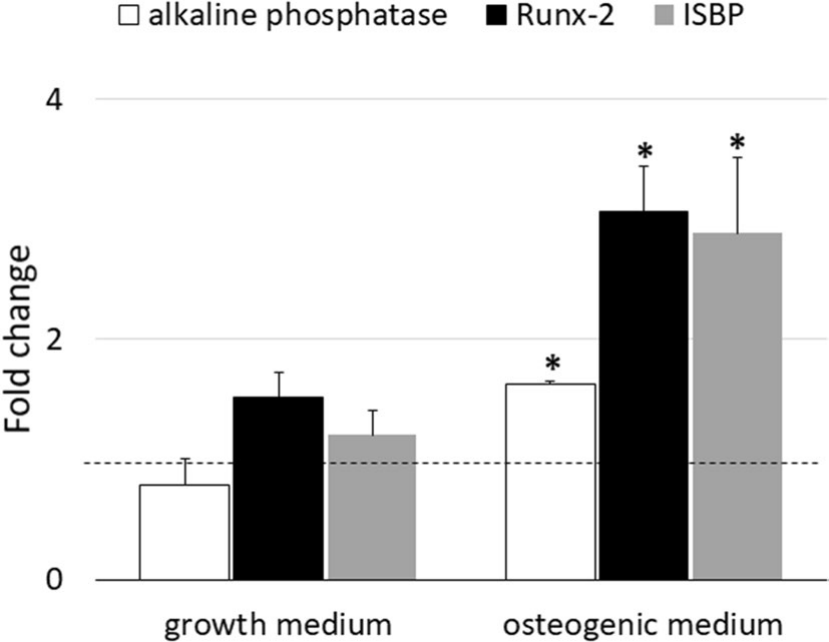
*alp*, *runx-2* and *ibsp* transcript expression in hASCs seeded on cage in the presence (OM) and absence (GM) of osteogenic factors after 14 days from differentiation. The values obtained were reported as ΔΔCT (fold change) versus 7 days expression (horizontal line). Each bar is the mean ± SEM of four different preparations. *, p ≤ 0.05 versus growth medium (Student t test).

mRNA expression of *alp*, *runx-2* and *ibsp* was assessed by RT-qPCR in hASCs seeded on cage cultured in growth and osteogenic media. According to the melt curve plot, there was only one peak corresponding to a single amplicon, indicating the specificity of the PCR reaction. Figure 4 shows results of *alp*, *runx-2* and *ibsp* mRNA expression at 14 days of differentiation expressed as fold change over hASCs at 7 days of culture in growth medium. After 14 days of culture in osteogenic medium, the expression level of the genes, was statistically higher than constructs cultured in growth medium: the expression of *alp* was twofold increased and the expression of *runx-2* and *ibsp* was almost threefold increased (Fig. 4).

#### Alkaline phosphatase activity

**Figure 5:**
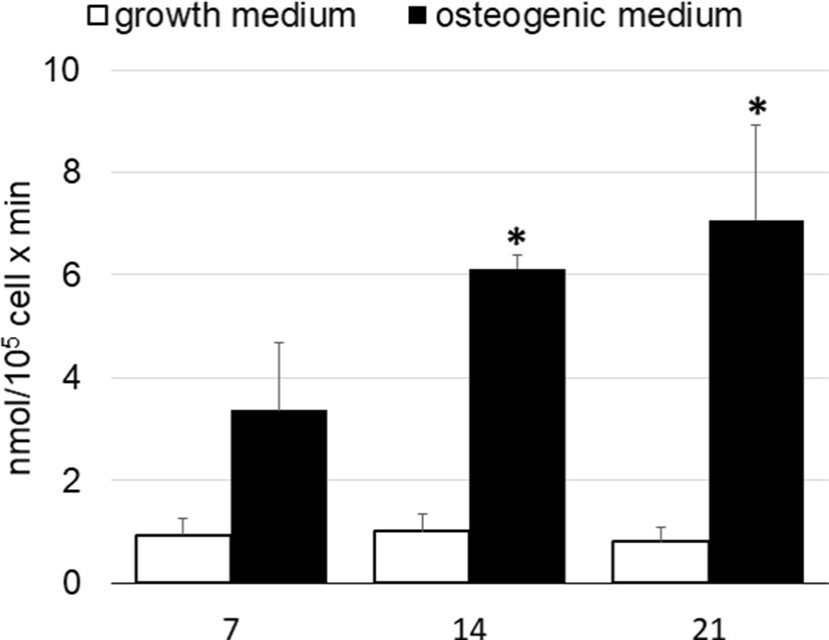
Alkaline phosphatase activity in hASCs grown on cage cultured with growth medium and osteogenic medium. Each bar represents the mean ± S.E.M. of four different preparations; each assay was repeated three times. *, p ≤ 0.05 vs. growth medium (one-way ANOVA method followed by Newman-Keuls’Q test).

To confirm the synthesis of functional proteins, alkaline phosphatase activity of cell lysates of hASCs/cage constructs was measured at days 7, 14 and 21 after differentiation. In accordance with increased alp mRNA expression, ALP activity increased in the presence of osteogenic medium. The increase was statistically significant at days 14 and 21 from differentiation (Fig. 5).

#### Calcium content

**Figure 6:**
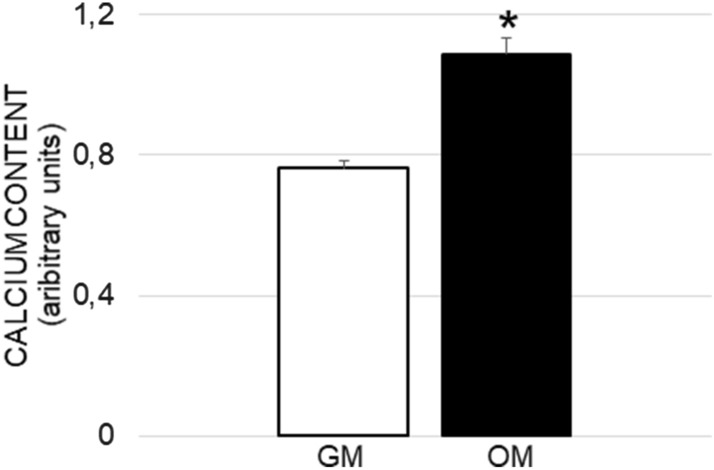
Calcium content in hASC/cage constructs cultured with growth (GM) and osteogenic (OM) medium. Each bar is the mean ± SEM of ten measurements. *, p ≤ 0.05 vs GM (Student t test).

The microanalysis performed with energy dispersive spectrometry (EDS) showed the presence of calcium on the hASCs/cage constructs cultured in growth and osteogenic media. On the constructs cultured in osteogenic medium the calcium content, expressed as arbitrary units, was statistically higher than in the constructs cultured in growth medium (Fig. 6).

#### Osteoinductive effects

**Figure 7:**
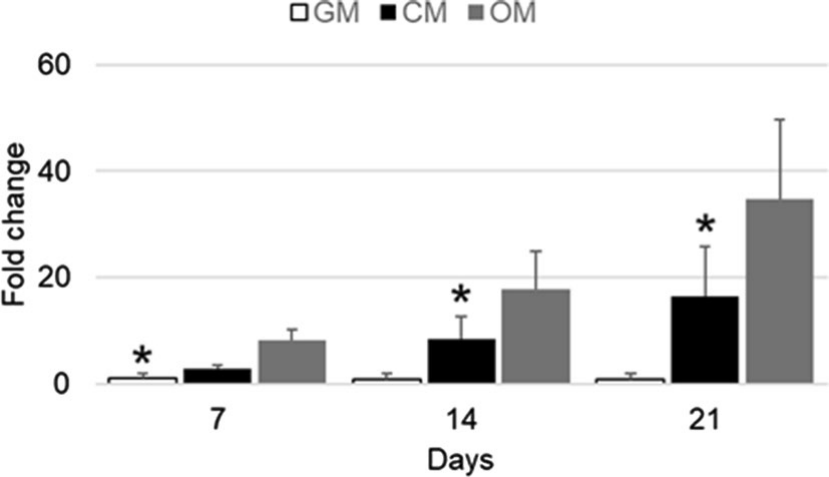
alp gene expression of hASCs grown in monolayer cultured with GM, CM and OM after 7, 14 and 21 days of in vitro culture. The values obtained were reported as ΔΔCT (fold change) versus 7 days expression. Each bar is the mean ± SEM of four different preparations. *, p ≤ 0.05 versus CM and OM (one-way ANOVA method followed by Newman-Keuls’Q test).

In order to evaluate the osteoinductive properties of titanium cages, cells grown in monolayer and cultured with GM, CM and OM were tested for their gene expression and enzymatic activity of ALP. The gene expression of *alp* was expressed as fold change over the hASC cultured in GM for 7 days. The cells cultured with CM displayed an increase in the gene expression level of alp both at 14 and 21 days of differentiation (p ≤ 0.05) compared to cells cultured with GM only (Fig. 7).

**Figure 8:**
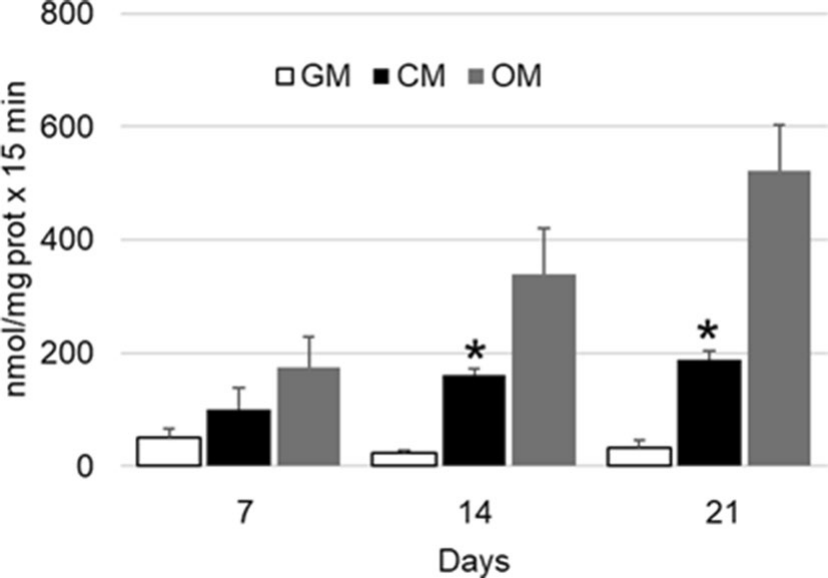
Alkaline phosphatase activity of hASCs grown in monolayer and cultured with GM, CM and OM at 7, 14 and 21 days of in vitro culture. Each bar represents the mean ± S.E.M. of four different preparations; each assay was repeated three times. *, p ≤ 0.05 vs. GM and OM (one-way ANOVA method followed by Newman-Keuls’Q test).

Moreover, to confirm the synthesis of the functional protein ALP, the alkaline phosphatase activity of cell lysates was measured at 7, 14 and 21 days of in vitro culture. As predicted by the qRT-PCR results, the alkaline phosphatase activity of cells cultured with CM showed a statistically significant increase of the enzymatic activity along the in vitro culture respect to cells grown in GM (Fig. 8).

## Discussion

Interbody fusion with different technical options is a very frequent and effective intervention for degenerative conditions of the cervical and lumbar spine. Main goal of the interbody fusion is to stabilize the spine with a solid arthrodesis, while correcting deformities and restoring the alignment. Among different technique of fusion, there are cage implants. An ideal cage design has to ensure the disc height and the correct alignment, achieving immediate postoperative stability to guarantee the osteointegration of the device and its long-term fixation, while reducing complication rates. Basic cage type is a small implant with lateral, upper and/or lower windows and a central cavity filled with either bone or osteo-inductive materials.

MT Ortho Company, using the electron beam melting (EBM) technology (MT Ortho s.r.l., Aci Sant’Antonio, CT, Italy), produced the cage tested in this in vitro study, with the following surface characteristics: two trabecular titanium endplates with a porosity of 80% and a pore size of 1 mm.

As shown by the SEM analysis, the cells seeded on the scaffold surfaces were able to migrate and colonize the inner portions of the cages. The production of an abundant extracellular matrix indicates that the material employed is cytocompatible and is able to favor cell adhesion and colonization, an appealing property in terms of biological behavior (Supplementary Fig. S1C, online).

The good cell adhesion and proliferation on the cage, evaluated by MTT assay, confirmed that the cell-material interaction is present and efficient, and that trabecular titanium represents a suitable material to be colonized, since the cell number increase was already statistically significant after 7 days from seeding. The growth was evaluated even at 14 and 21 days of culture, showing that hASCs kept on dividing and colonizing the cage (Fig. 1).

Previous studies in literature demonstrated that trabecular titanium displays osteoinductive effects, meaning that the physical and chemical properties of the material are able to commit undifferentiated cells toward an osteogenic phenotype^17^. Therefore, in order to evaluate the osteoinductive cability of MT Ortho scaffolds, we seeded cells on the cages, cultured them both into growth and osteogenic media and then assessed the osteogenic differentiation by analyzing bone specific markers.

We investigated the gene expression level of *alp*, *runx-2* and *ibsp*, bone specific genes. RUNX-2 is a precocious transcription factor required for the commitment toward the osteoblastic lineage, hence representing an excellent marker for the initiation of osteogenic differentiation; the multifunctional enzyme ALP, instead, is a marker of bone matrix mineralization. Finally, bone sialoprotein (IBSP) is a marker of bone extracellular matrix. Moreover, we evaluated the cellular content of the functional protein ALP to confirm the qRT-PCR data already collected.

The results obtained by quantitative gene expression analysis indicate that the hASC were able to differentiate into osteoblast like cells when cultured in presence of osteogenic factors (Fig. 4). Interestingly, also the gene expression of *runx-2* and *ibsp* of hASC cultured in growth medium for 14 days was increased respect to that of cells cultured in growth medium for 7 days (horizontal line, Fig. 4). Therefore, a deeper investigation was carried out: the hASCs were cultured in monolayer with GM, CM and OM for 21 days and tested for the gene expression and enzymatic activity of alkaline phosphatase at 7, 14 and 21 days of culture. The qRT-PCR results indicated that cells cultured with CM displayed a significant increase (p ≤ 0.05) of *alp* expression respect to control, already at 7 days of in vitro culture (Fig. 7). However, the difference between the gene expression of cells grown in CM and OM, even though present, was not statistically significant, suggesting that a conspicuous number of hASCs may have entered the osteogenic differentiation pathway. The results were confirmed by the analysis of the ALP activity, that was statistically higher in cells cultured with CM than in control (GM) (Fig. 8). These results, taken together, suggest that the cages utilized were able to trigger a change toward the osteogenic differentiation even in absence of osteogenic factors, because of soluble factors and microvesicles secreted by the hASCs seeded on the scaffolds^18^. In literature has been reported that the biomaterials must meet very specific requirements in terms of macrostructure (like as geometry and porosity), microstructure (such as microporosity and surface roughness) and chemical composition in order to be osteoinductive^19^. Even though the molecular mechanism of osteoinduction is still largely unknown^20^, the understanding of parameters affecting the osteoinductivity of biomaterials represents a gold starting point in order to develop efficient devices able to interact positively with the host tissue environment, enhancing tissue repair and regeneration.

To conclude, the trabecular titanium cage used in this study has a sufficient roughness to allow attachment, migration and proliferation of hASCs without any surface treatments, intended to improve the biological behavior of the scaffold. Further, our results demonstrated that the hASCs seeded on TT, were able to produce a secretome enriched with molecules able to trigger a shift in the hASCs phenotype, towards the osteogenic differentiation. Other authors demonstrated that highly porous Ti, without any surface treatment, was able to trigger cellular proliferation, colonization and differentiation toward the osteoblastic lineage^21,22^. Moreover, trabecular titanium also allows the adhesion and proliferation of endothelial progenitor cells and, consequently, by permitting vascularization, favors osteointegration^23^.

## Conclusions

In conclusion, these in vitro studies on highly porous trabecular titanium cage, manufactured by the EBM technology, showed that the hASCs could adhere, migrate and proliferate without the necessity to modify either chemically or physically the scaffold’s surface.

Moreover, the specific cage design displayed the ability to trigger the osteogenic differentiation, a gold starting point in the comprehension of osteoinduction mechanism. In terms of biological behavior, highly porous metal cages made by EBM technology, similar to the trabecular bone, might offer a great capacity for osteoinduction and a good potential for bone regeneration, as suggested by the results of these in vitro studies. However, despite the promise of these scaffolds, an in vivo investigation about osteointegration and osteoinduction capabilities is required.

## Supplementary information

Supplementary Information.

## References

[1] Chau AMT, Mobbs RJ (2009). Bone graft substitutes in anterior cervical discectomy and fusion. Eur. Spine J..

[2] Jain S, Eltorai AEM, Ruttiman R, Daniels AH (2016). Advances in spinal interbody cages. Orthop. Surg..

[3] Cloward RB (2007). The anterior approach for removal of ruptured cervical disks. J. Neurosurg. Spine.

[4] McConnell, J.R., Freeman, B.J.C., Debnath, U.K., Grevitt, M.P., Prince, H.G., Webb, J.K. A prospective randomized comparison of coralline hydroxyapatite with autograft in cervical interbody fusion. *Spine (Phila. Pa. 1976)* **28** (2003). 10.1097/01.BRS.0000048503.51956.E1 12590203

[5] Bagby GW (1988). Arthrodesis by the distraction-compression method using a stainless steel implant. Orthopedics.

[6] Hacker RJ (2000). A randomized prospective study of an anterior cervical interbody fusion device with a minimum of 2 years of follow-up results. J. Neurosurg..

[7] Kettler, A.; Claes, L. Primary stabilizing effect of interbody fusion devices for the cervical spine : An in vitro comparison between three different cage types and bone cement. 410–416 (2000). 10.1007/s005860000168 PMC3611385 11057535

[8] Benzel, C. E. *Spine Surgery: Techniques, Complication Avoidance, and Management* (Elsevier, Ed.; 2nd ed. 2005; ISBN 0443066167).

[9] Matsumoto T, Okuda S, Maeno T, Yamashita T, Yamasaki R, Sugiura T, Iwasaki M (2017). Spinopelvic sagittal imbalance as a risk factor for adjacent-segment disease after single-segment posterior lumbar interbody fusion. J. Neurosurg. Spine.

[10] Fountas, K.N., Kapsalaki, E.Z., Nikolakakos, L.G., Smisson, H.F., Johnston, K.W., Grigorian, A.A., Lee, G.P., Robinson, J.S.J. Anterior cervical discectomy and fusion associated complications. *Spine (Phila. Pa. 1976).* **32**, 2310–2317 (2007). 10.1097/BRS.0b013e318154c57e 17906571

[11] Hanc M, Fokter SK, Vogrin M, Molicnik A, Recnik G (2016). Porous tantalum in spinal surgery: An overview. Eur. J. Orthop. Surg. Traumatol..

[12] Chong E, Pelletier MH, Mobbs RJ, Walsh WR (2015). The design evolution of interbody cages in anterior cervical discectomy and fusion: A systematic review orthopedics and biomechanics. BMC Musculoskelet. Disord..

[13] Siu TL, Rogers JM, Lin K, Thompson R, Owbridge M (2018). Custom-made titanium 3-dimensional printed interbody cages for treatment of osteoporotic fracture-related spinal deformity. World Neurosurg..

[14] Saler, M., Caliogna, L., Botta, L., Benazzo, F., Riva, F., Gastaldi, G. *hASC and DFAT , Multipotent Stem Cells for Regenerative Medicine : A Comparison of Their Potential Differentiation In Vitro*. (2017) 10.3390/ijms18122699 PMC5751300 29236047

[15] Gastaldi G, Asti A, Scaffino MF, Visai L, Saino E, Cometa AM, Benazzo F (2010). Human adipose-derived stem cells (hASCs) proliferate and differentiate in osteoblast-like cells on trabecular titanium Scaffolds. J. Biomed. Mater. Res. A..

[16] Laforenza U, Miceli E, Gastaldi G, Scaffino MF, Ventura U, Fontana JM, Orsenigo MN, Corazza GR (2010). Solute transporters and aquaporins are impaired in celiac disease. Biol. Cell.

[17] Albrektsson T, Johansson C (2001). Osteoinduction, osteoconduction and osseointegration. Eur. Spine J..

[18] Linero I, Chaparro O (2014). Paracrine effect of mesenchymal stem cells derived from human adipose tissue in bone regeneration. PLoS ONE.

[19] Barradas AMC, Yuan H, van Blitterswijk CA, Habibovic P (2011). Osteoinductive biomaterials: Current knowledge of properties, experimental models and biological mechanisms. Eur. Cell. Mater..

[20] Habibovic P, Sees TM, van den Doel MA, van Blitterswijk CA, de Groot K (2006). Osteoinduction by biomaterials—Physicochemical and structural influences. J. Biomed. Mater. Res. Part A.

[21] Tamaddon, M., Samizadeh, S., Wang, L., Blunn, G., Liu, C. Intrinsic osteoinductivity of porous titanium Scaffold for bone tissue engineering. *Int. J. Biomater.* (2017). 10.1155/2017/5093063 PMC5549492 28814954

[22] Benazzo F, Botta L, Scaffino MF, Caliogna L, Marullo M, Fusi S, Gastaldi G (2014). Trabecular titanium can induce in vitro osteogenic differentiation of human adipose derived stem cells without osteogenic factors. J. Biomed. Mater. Res. Part A.

[23] Gastaldi G, Caliogna L, Botta L, Ghiara M, Benazzo F (2015). Endothelial progenitor cell adhesion, growth and characterization on trabecular titanium and trabecular titanium coated with collagen or decellularized ECM. J. Biol. Regul. Homeost. Agents.

