# Supplementary material for: Osteogenic potential of human adipose derived stem cells (hASCs) seeded on titanium trabecular spinal cages
Source: Sci Rep. 2020 Oct 26;10:18284. doi: 10.1038/s41598-020-75385-y (PMC7589498; doi:10.1038/s41598-020-75385-y)

Osteogenic potential of human adipose derived stem cells (hASCs) seeded on titanium trabecular spinal cages

Caliogna Laura[1#], Bina Valentina[5#], Botta Laura[2], Benazzo Francesco Maria[1,4], Medetti Marta[1], Maestretti Gianluca[7], Mosconi Mario[1], Cofano Fabio[8,9*], Tartara Fulvio Alberto[6], Gastaldi Giulia[4,5]

[1], Orthopedics and Traumatology Clinic, IRCCS Policlinico San Matteo Foundation, 27100 Pavia, Italy

[2], Department of Biology and Biotechnology "Lazzaro Spallanzani", University of Pavia, 27100 Pavia, Italy

[3], Department of Clinical Surgical, Diagnostic and Pediatric Sciences, University of Pavia, 27100 Pavia, Italy

[4], Centre for Health Technologies, University of Pavia, 27100 Pavia, Italy

[5], Department of Molecular Medicine, University of Pavia, 27100 Pavia, Italy

[6], Istituto Clinico Città Studi, Milan, Italy

[7], Department of Orthopaedic Surgery, Cantonal Hospital, 1708 Fribourg, Switzerland

[8], Department of Neuroscience "Rita Levi Montalcini", Unit of Neurosurgery, 10126 Turin, Italy

[9], Spine Surgery Unit, Humanitas Gradenigo, Turin, Italy

[#], Contributed equally

*, Correspondence: Fabio Cofano, E-mail: fabio.cofano@gmail.com

Department of Neuroscience "Rita Levi Montalcini", Unit of Neurosurgery, 10126 Turin, Italy

MD, E-mail: fabio.cofano@gmail.com

Received: date; Accepted: date; Published: date

Supplementary figures

Supplementary Figure S1. Scansion electron microscopy of empty cage (A) and of cage seeded with hASCs and cultured in growth medium (B) and osteogenic medium (C) for 21 days. Magnifications: A: 202x, B: 205x; C: 202x

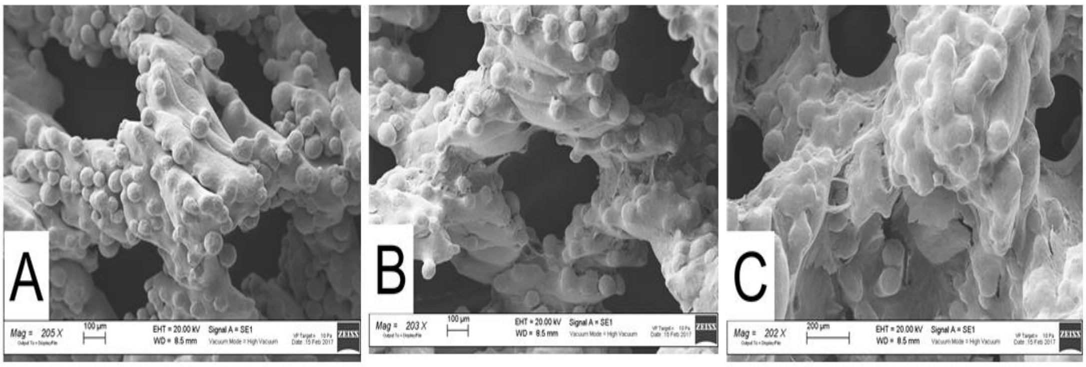

Supplementary Figure S2. Scansion electron microscopy of the internal of the cage. (A) Empty cage; (B) cage seeded with hASCs and cultured in growth medium and (C) cultured in osteogenic medium for 21 day Magnifications: A: 205x, B: 203x; C: 202x.

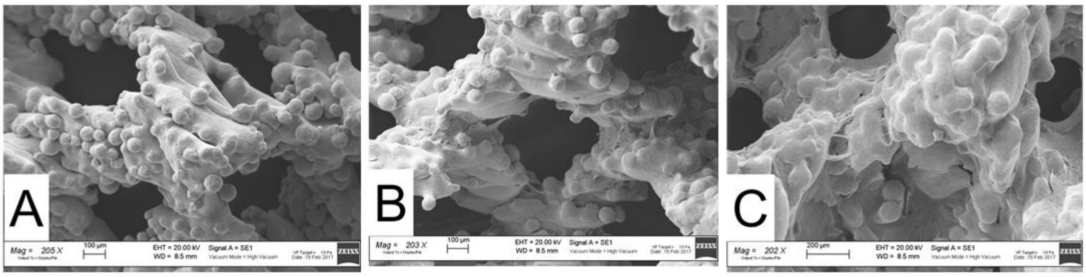

Supplement: Supplementary file 1 — Supplementary Information. [file 41598_2020_75385_MOESM1_ESM.pdf]
